# Free energy calculations shed light on the nuclear pore complex’s selective barrier nature

**DOI:** 10.1016/j.bpj.2021.07.025

**Published:** 2021-07-31

**Authors:** Atsushi Matsuda, Mohammad R.K. Mofrad

**Affiliations:** 1 Molecular Cell Biomechanics Laboratory, Departments of Bioengineering and Mechanical Engineering, University of California Berkeley, Berkeley, California; 2 Molecular Biophysics and Integrative Bioimaging Division, Lawrence Berkeley National Laboratory, Berkeley, California

## Abstract

The nuclear pore complex (NPC) is the exclusive gateway for traffic control across the nuclear envelope. Although smaller cargoes (less than 5–9 nm in size) can freely diffuse through the NPC, the passage of larger cargoes is restricted to those accompanied by nuclear transport receptors (NTRs). This selective barrier nature of the NPC is putatively associated with the intrinsically disordered, phenylalanine-glycine repeat-domains containing nucleoporins, termed FG-Nups. The precise mechanism underlying how FG-Nups carry out such an exquisite task at high throughputs has, however, remained elusive and the subject of various hypotheses. From the thermodynamics perspective, free energy analysis can be a way to determine cargo’s transportability because the traffic through the NPC must be in the direction of reducing the free energy. In this study, we developed a computational model to evaluate the free energy composed of the conformational entropy of FG-Nups and the energetic gain associated with binding interactions between FG-Nups and NTRs and investigated whether these physical features can be the basis of NPC’s selectivity. Our results showed that the reduction in conformational entropy by inserting a cargo into the NPC increased the free energy by an amount substantially greater than the thermal energy (≫*k*_B_*T*), whereas the free energy change was negligible (<*k*_B_*T*) for small cargoes (less than ~6 nm in size), indicating the size-dependent selectivity emerges from the entropic effect. Our models suggested that the entropy-induced selectivity of the NPC depends sensitively upon the physical parameters such as the flexibility and the length of FG-Nups. On the other hand, the energetic gain via binding interactions effectively counteracted the entropic reduction, increasing the size limit of transportable cargoes up to the nuclear pore size. We further investigated the geometric effect of the binding spot spatial distribution and found that the clustered binding spot distribution decreased the free energy more efficiently as compared to the scattered distribution.

## Significance

This study evaluated the change in free energy caused by the cargo insertion into the NPC and showed that the free energy change increased significantly with the cargo size, demonstrating the size-dependent selectivity stemming from the entropic barrier effect of FG-Nups. We calculated the free energy for each cargo size and estimated the critical cargo diameter, which yields the free energy difference being equal to the thermal energy *k*_B_*T*. The binding interactions between FG-Nups and nuclear transport receptors effectively lowered the free energy and increased the critical cargo diameter. We further investigated the role of the binding spot distribution and found that the clustered binding spot decreases the free energy efficiently.

## Introduction

The nuclear pore complex (NPC) is a protein assembly that perforates the nuclear envelope, creating an exclusive gateway for nucleocytoplasmic transport (1, 2, 3, 4, 5). The NPC acts as a selective barrier, controlling the molecular passage across the nuclear envelope. There are two different ways of the molecular transport through the NPC, namely the passive diffusion and the facilitated transport. The passive diffusion is the way that molecules randomly translocate through the NPC by their Brownian motion, and it is restricted to small molecules being less than 5–9 nm in size (6, 7, 8). The facilitated transport occurs when molecules are bound to the nuclear transport receptors (NTRs), which can interact with the NPC to enhance the molecular translocation, and the size limit of the molecule is much larger than the passive diffusion, up to ~39 nm (9). Because only small molecules can adopt the passive diffusion, whether or not molecules are bound to NTRs is key to determining the translocations of large molecules. Hereafter, we refer to the molecule-NTR complex as “attractive cargo” and the one without NTRs as “inert cargo.” The NPC exquisitely distinguishes the attractive and inert cargoes with a high accuracy, ensuring vigorous separation between the nucleoplasm and the cytoplasm.

The NPC recognizes and interacts with NTRs using FG nucleoporins (FG-Nups), which are molecular components of the NPC comprising its central channel. The FG-Nups are intrinsically disordered proteins (10), i.e., they lack any folded secondary structures and can change their conformations dynamically. One end of the FG-Nup is tethered to the channel wall, whereas the other end is not connected to the structure dangling freely inside the nuclear pore. The FG-Nups contain phenylalanine- and glycine-rich motifs such as FG, GLFG, and FxFG (11, 12, 13), and through these motifs, they can weakly bind with NTRs via hydrophobic interactions. Because the FG-Nups are spatially exposed to where inert or attractive cargoes translocate through the NPC, they have direct access to the cargoes to either promote or hinder their passage.

The details of how FG-Nups create a barrier while selectively allowing attractive cargoes to pass through the nuclear pore remains elusive. Several models have been proposed to explain the NPC’s selective barrier nature (14,15). The virtual-gate model, for example, suggests that a reduction in the conformational entropy of FG-Nups leads to the blockage of inert cargoes. Because FG-Nups are intrinsically disordered and can flexibly change their structures, the total number of their realizable structures, i.e., their conformational entropy, is large in general. However, as the cargo translocates through the NPC, it reduces the space available to FG-Nups, limiting their obtainable shapes and reducing their conformational entropy. When the cargo size is large enough, the entropic reduction becomes non-negligible, and that virtually prevents the passage of inert cargoes. On the other hand, attractive cargoes can hydrophobically bind with FG-Nups stabilizing the energetic landscape, which can counteract the entropic effect and facilitate their transport through the NPC. This idea can be thermodynamically expressed using the free energy *F* = *E* − *TS*, where *E*, *T*, and *S* are the energy, absolute temperature, and entropy of the system, respectively. The cargo’s entry into the nuclear pore reduces the entropy *S*, and the binding interactions between NTRs and FG-Nups reduces the energy *E*. When the total change in free energy Δ*F* is less than the thermal energy *k*_B_*T*, where *k*_B_ is the Boltzmann constant, the cargo can pass through the NPC without any external input, such as the mechanical force pushing the cargo into the pore, except the thermal fluctuation.

The entropic barrier hypothesis for NPC’s selectivity, or the virtual-gate model, has been investigated extensively. Electron microscopy studies (16, 17, 18, 19, 20, 21) showed that the central plug, a putative structure in the middle of the NPC channel, was often vague or undefined, implying the multiplicity of FG-Nups’ conformation. This was further confirmed when the computational structure estimation demonstrated many different possible conformations of FG-Nups (22,23). At the submolecular scale, atomic force microscopy (24, 25, 26) and NMR (27,28) experiments provided data on the flexibility and noncohesiveness of FG-Nups, essential features to create the entropic barrier. From the dynamical point of view, atomic force microscopy measurements (29) showed that FG-Nups fluctuate rapidly inside the nuclear pore. Molecular dynamics (30,31), Langevin dynamics (32, 33, 34, 35, 36), and Brownian dynamics simulations (37, 38, 39, 40, 41) demonstrated the highly dynamic nature of FG-Nups. However, some experimental studies argued that the entropic effect by itself is too weak to account for the mechanical stiffness in the NPC central channel (42,43). Another model explaining the NPC’s barrier formation is the selective phase model (44,45), which assumes that FG-Nups are interconnected, creating a meshwork inside the pore functioning as a molecular sieve. The selective phase model suggests the physical meshwork, rather than the entropic effect, creates the barrier in the pore. This model was well validated by examining the physical properties of the hydrogel composed of mutually cross-linked FG-Nups (46, 47, 48). Now it is generally considered that both of those effects, i.e., the entropic exclusion and the physical meshwork connections, contribute to the NPC’s selective barrier formation (49,50).

The aim of this study was to explore the validity of the entropic barrier hypothesis quantitatively by constructing a computational model of NPC’s free energy. To investigate the relation between the entropic barrier effect and the NPC’s selective nature, we modeled the free energy by specifically considering the conformational entropy of FG-Nups and the binding interaction between FG-Nups and NTRs. Other groups have previously proposed the models for the NPC’s free energy using the molecular theory (51, 52, 53, 54, 55), the density functional theory (56,57), and umbrella sampling of the molecular dynamics simulation trajectories (33,36). Our model is different from prior art in two ways: firstly, we assumed that FG-Nups are homogeneous ideal chains instead of considering their amino acid sequence heterogeneity (33,36,52,54) or intermolecular cohesiveness (56,57). By this reductionist approach, we focused on studying the effect of FG-Nups’ conformational entropy in absence of other physical factors. Secondly, we modeled FG-Nups as continuous Gaussian chains. In contrast to the rotational isomeric state description of FG-Nups (51,53,55), the Gaussian chain model explicitly includes the Kuhn length in its formulation, which enabled us to study the effect of FG-Nups’ flexibility on the entropic barrier. Also, because the Gaussian chain model handles the microscopic elasticity along polymer’s backbone, it effectively captured the fine details in surface geometry of the domain boundary, which produced one of the important results of this work, the effect of the binding spot distribution.

Using our computational model, we analyzed the change in free energy when inert or attractive cargoes enter the NPC. Our result showed that the entropic effect alone can effectively create the size-dependent selectivity and that the interaction between NTRs and FG-Nups largely shifted the size limit of transportable cargoes. We then investigated how the physical properties of FG-Nups, such as their Kuhn length and total length, and the geometry of the NPC change the behavior of the entropic barrier. Finally, we studied how the total number and the surface distribution of the binding spots on attractive cargoes contribute to reducing the free energy of the system. Our study sheds more light on the physical foundation of NPC’s selectivity and also offers a platform for custom design and shape configuration of drug-delivering cargoes targeted for the nucleus (58, 59, 60) and ultimately inspires NPC-mimetic filtering systems for industrial applications (61, 62, 63, 64, 65, 66).

## Materials and methods

To quantitatively analyze the entropic barrier effect of the NPC, we mathematically modeled the NPC system comprising FG-Nups and the inert or attractive cargo located inside the nuclear pore. We modeled the system’s free energy based on Edward’s field theory of polymers (67, 68, 69), which is used for the analysis of flexible polymers. Edward’s theory models the free energy using field variables instead of explicitly describing three-dimensional curves of each polymer, which makes the calculation analytically or computationally tractable. Considering the ability of this model to efficiently describe the biological systems (70, 71, 72, 73, 74, 75), we used it to study the physical features of the NPC.

### Formulation of the free energy

We modeled FG-Nups, the intrinsically disordered FG repeat-domains containing nucleoporins, as continuous Gaussian chains, i.e., linear polymers having elastic bonds (67, 68, 69). Each FG-Nup was represented as a spatial curve ***R***(*s*), with *s* ∈ [0, *N*] being a contour position along the chain’s backbone. The upper limit of the contour position *N* was determined as *N* = *l*/*b*, where *l* is the length of FG-Nups and *b* is their Kuhn length. The potential energy associated with each configuration ***R***(*s*) is written as

(1) $$U_{0}\left[R\right]=\frac{3k_{\text{B}}T}{2b^{2}}\int_{0}^{N}ds{\left|\frac{dR\left(s\right)}{ds}\right|}^{2},$$

where *k*_B_ is the Boltzmann constant and *T* is the absolute temperature. The conformational entropy effects are included through the term *U*_0_[***R***].

Upon the existence of an external field *V*(***r***) acting on each segment located at ***r***, an additional potential energy emerges as

(2) $$U_{1}\left[R\right]=\int_{0}^{N}dsV\left(R\left(s\right)\right).$$

In our model, *V*(***r***) represents an adsorption field around the attractive cargo, and thus, *U*_1_[***R***] corresponds to the energetic gain by the bindings between FG-Nups and the NTR.

Suppose there are *n* independent FG-Nups each having a conformation of ***R***_*i*_ (*i* = 1, 2, …, *n*), whose starting and ending positions are ***R***_*i*_(0) = $r_{⊥,i}$ and ***R***_*i*_(*N*), then the partition function of the system can be written as

(3) $$Z=\overset{n}{\underset{i=1}{\prod }}\underset{\text{Ω}}{\int }dr\int_{R_{i}\left(0\right)=r_{⊥,i}}^{R_{i}\left(N\right)=r}DR_{i}\text{exp}\left(-\beta U_{0}\left[R_{i}\right]-\beta U_{1}\left[R_{i}\right]\right),$$

where *β* ≡ 1/(*k*_B_*T*) is the inverse of the thermal energy. The integral $\int DR_{i}$ indicates a functional integral, which is carried out over all possible conformation curves ***R***_*i*_ (67). The notation Ω means that the spatial integral ∫dr is carried out in a particular domain of interest. In this formulation, interactions between non-neighboring segments of FG-Nups are ignored. The free energy of the system *F* is calculated using the partition function *Z*, such that

(4) $$F=-\frac{1}{\beta }\text{ln}Z.$$

Carrying out the functional integral of Eq. 3 explicitly is cumbersome, so to make the calculation more manageable, we introduced the Green function

(5) $$G\left(r,r^{\text{′}},s\right)\equiv \int_{R\left(0\right)=r}^{R\left(s\right)=r^{\text{′}}}DR\text{exp}\left(-\beta U_{0}\left[R\right]-\beta U_{1}\left[R\right]\right),$$

which signifies the statistical weight of an FG-Nup whose starting and ending positions are ***R***(0) = ***r*** and ***R***(*s*) = ***r***′. We define the conditions for the Green function when *s* ≤ 0, such that

(6) $$G\left(r,r^{\text{′}},s\right)=0\;\text{for}\;s<0,\;G\left(r,r^{\text{′}},0\right)=\delta \left(r-r^{\text{′}}\right).$$

With this definition, the Green function *G*(***r***, ***r***′, *s*) satisfies the modified diffusion equation (MDE) below (see Supporting materials and methods and (67,68) for the derivation):

(7) $$\left[\frac{\partial }{\partial s}-\frac{b^{2}}{6}\nabla_{r}^{2}+\beta V\left(r\right)\right]G\left(r,r^{\text{′}},s\right)=0,$$

where the differential operator $\nabla_{r}$ acts on the spatial coordinate ***r***. Using the Green function *G*(***r***, ***r***′, *s*), the partition function *Z* is expressed such that

(8) $$Z=\overset{n}{\underset{i=1}{\prod }}\underset{\text{Ω}}{\int }drG\left(r_{⊥,i},r,N\right),$$

where $r_{⊥,i}$ is the tethered position of an FG-Nup *i*. It should be noted that Eq. 8 does not explicitly include the functional integral, making the calculation of *Z* more manageable. Every geometrical restriction represented by the functional integral is incorporated in the MDE; thus, Eqs. 7 and 8 are equivalent to Eq. 3.

### Mean segment density distribution

To discuss the geometrical effect of the boundary conditions on system’s free energy, we calculated the spatial distribution of the mean segment density *ρ*(***r***). The mean segment density *ρ*(***r***) is the ensemble average of the microscopic segment density $\hat{\rho }$(***r***, {[***R***_*i*_]_*i* = 1…*n*_}) over all possible conformations of FG-Nups. We defined the microscopic segment density $\hat{\rho }$(***r***, {[***R***_*i*_]_*i* = 1…*n*_}) such that

(9) $$\hat{\rho }\left(r,\left\{{\left[R_{i}\right]}_{i=1...n}\right\}\right)=\overset{n}{\underset{i=1}{\sum }}{\hat{\rho }}_{i}\left(r,\left[R_{i}\right]\right)\equiv \overset{n}{\underset{i=1}{\sum }}\int_{0}^{N}ds\delta \left(r-R_{i}\left(s\right)\right).$$

The mean segment density *ρ*(***r***) can be calculated using the Green function *G*(***r***, ***r***′, *s*), which we introduced in the previous section (see Supporting materials and methods and (69) for the derivation),

(10) $$\rho \left(r\right)=\hat{\rho }\left(r,\left\{{\left[R_{i}\right]}_{i=1...n}\right\}\right)$$

$$=\overset{n}{\underset{i=1}{\sum }}\frac{\int_{0}^{N}ds\int_{\text{Ω}}dr^{\text{′}}G\left(r_{⊥,i},r,s\right)G\left(r,r^{\text{′}},N-s\right)}{\int_{\text{Ω}}dr^{\text{′}}G\left(r_{⊥,i},r^{\text{′}},N\right)}.$$

### Calculation procedure

To proceed with our calculation, we introduced the following functions:

(11) $$\tilde{q}\left(r,s\right)\equiv \underset{\text{Ω}}{\int }dr^{\text{′}}G\left(r^{\text{′}},r,s\right),\;q_{\text{c}}\left(r,s\right)\equiv \overset{n}{\underset{i=1}{\sum }}\frac{G\left(r_{⊥,i},r,s\right)}{\tilde{q}\left(r_{⊥,i},N\right)}.$$

The partition function and the mean segment density can be expressed using $\tilde{q}$(***r***, *s*) and *q*_c_(***r***, *s*) such that

(12) $$Z=\overset{n}{\underset{i=1}{\prod }}\tilde{q}\left(r_{⊥,i},N\right)$$

(13) $$\rho \left(r\right)=\int_{0}^{N}dsq_{\text{c}}\left(r,s\right)\tilde{q}\left(r,N-s\right).$$

Because $\tilde{q}$(***r***, *s*) and *q*_c_(***r***, *s*) are linear combinations of the Green function *G*(***r***, ***r***′, *s*), they satisfy the MDE,

(14) $$\left[\frac{\partial }{\partial s}-\frac{b^{2}}{6}\nabla_{r}^{2}+\beta V\left(r\right)\right]q\left(r,s\right)=0,$$

where *q*(***r***, *s*) denotes either $\tilde{q}$(***r***, *s*) or *q*_c_(***r***, *s*). The initial conditions for $\tilde{q}$(***r***, *s*) and *q*_c_(***r***, *s*) are

(15) $$\tilde{q}\left(r,0\right)=1,\;q_{\text{c}}\left(r,0\right)=\overset{n}{\underset{i}{\sum }}\frac{\delta \left(r-r_{⊥,i}\right)}{\tilde{q}\left(r_{⊥,i},N\right)}.$$

Our calculation pipeline is as follows: we first calculated $\tilde{q}$(***r***, *s*) by solving the MDE (Eq. 14) with the initial condition (Eq. 15) and the boundary condition explained in the section below. Then, using the value $\tilde{q}$(***r***, *N*) as an input for the initial condition of *q*_c_(***r***, *s*) (Eq. 15), we calculated *q*_c_(***r***, *s*) by solving the MDE (Eq. 14) with the same boundary condition. Finally, we calculated the free energy (Eqs. 4 and 12) and the mean segment density (Eq. 13). The parameters used for our calculation are summarized in Table 1.

**Table 1:** Mechanical and geometrical parameters of the NPC

Symbol	Value	Unit	Description	Reference
*n*	80	–	number of FG-Nups	(76)^a^
*b*	0.86	nanometers	Kuhn length of FG-Nups	(24)
*l*	180	nanometers	total length of FG-Nups	(50)^b^
*D*_pore_	40	nanometers	diameter of NPC channel	(76)^a^
*h*_pore_	40	nanometers	height of NPC channel	(76)^a^

Listed here are the reference values, which we used throughout this study if not specified otherwise.

a The geometry of the yeast NPC is used.

b *l* is set to be the average length of FG-Nups.

### Boundary conditions

**Figure 1:**
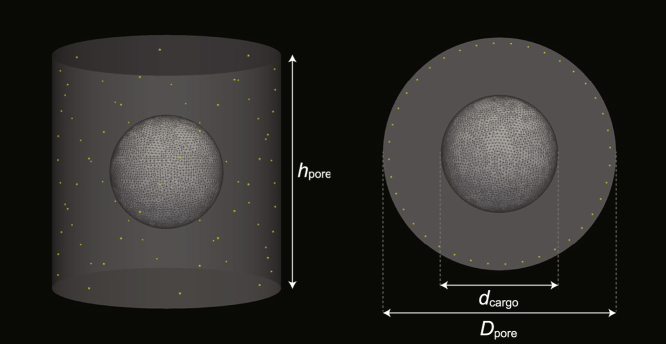
Geometry of the calculation domain modeling the NPC; side view (*left*) and top view (*right*). The nuclear pore was modeled as a cylinder of diameter *D*_pore_ and height *h*_pore_. The cargo was modeled as a sphere of diameter *d*_cargo_ located at the center of the pore. Tethered positions of FG-Nups were obtained from the reconstructed structure of the NPC (22,23) and marked as yellow dots here.

We calculated the MDE in a three-dimensional domain that models the NPC’s central channel (Fig. 1). The geometry of the NPC was modeled as a cylinder of diameter *D*_pore_ and height *h*_pore_ (76). The two open ends of the NPC, i.e., the top and bottom faces of the cylinder, were set to be open boundaries, and the inner wall, corresponding to the cylinder side, was modeled to be the hard surface. We also added a domain boundary that models an inert or attractive cargo inside the nanopore (Fig. 1) to study the effect of the cargo insertion into the NPC. The cargo was modeled as a sphere of diameter *d*_cargo_ having the hard wall surface. In this study, we calculated the free energy with and without a cargo inside the NPC, indicated by *F*_cargo_ and *F*_empty_, respectively, and compared their difference. When we calculated *F*_cargo_, we set cargo’s position at the center of the NPC except the case of Fig. S4.

The hard surfaces, i.e., the NPC’s inner wall and the cargo’s surface, were modeled as the Dirichlet boundary conditions,

(16) $$\tilde{q}\left(r_{\text{D}},s\right)=0,\;q_{\text{c}}\left(r_{\text{D}},s\right)=0,$$

where ***r***_D_ ∈ ∂Ω_D_ (∂Ω_D_ is the Dirichlet boundary). The two open ends of the NPC were modeled as the Neumann boundaries,

(17) $$\frac{\partial \tilde{q}\left(r_{\text{N}},s\right)}{\partial n}=0,\;\frac{\partial q_{\text{c}}\left(r_{\text{N}},s\right)}{\partial n}=0,$$

where ***r***_N_ ∈ ∂Ω_N_ (∂Ω_N_ is the Neumann boundary) and ***n*** is the vector perpendicular to the boundary surface.

Tethered positions of FG-Nups $r_{⊥,i}$ (*i* = 1, 2, …, *n*), which we used to calculate the partition function and the initial condition of *q*_c_(***r***, *s*), were determined according to the estimated structure of the NPC (22,23). To avoid numerical errors stemming from the spatial discretization, we relocated all tethered positions 1 nm inward from the channel wall (Fig. 1).

### External potential

The interaction between FG-Nups and the NTR was modeled by the potential term *V*(***r***). We set binding spots ***r***_bind, *i*_ (*i* = 1, 2, …, *N*_bind_) on the surface of the cargo, where *N*_bind_ is the total number of the binding spots, and defined the potential as

(18) $$V\left(r\right)=\left\{ \begin{array}{ll} -\gamma & \text{min}\left|r-r_{\text{bind},i}\right|<r_{\text{cutoff}}\\ 0 & \text{otherwise} \end{array}\right.$$

where *γ* is the interfacial energy (77), min|***r*** − ***r***_bind, *i*_| is the minimal distance between the position ***r*** and ***r***_bind, *i*_ (*i* = 1, 2, …, *N*_bind_), and *r*_cutoff_ is the cutoff distance. The interfacial energy *γ* was explored from 0.9 to 1.2 *k*_B_*T*, which is in the reasonable range to model the weak interaction between FG-Nups and NTRs (78). The cutoff distance *r*_cutoff_ was set to be equal to the reference Kuhn length *b* = 0.86 nm following the model by Yang et al. (70).

The binding spots ***r***_bind, *i*_ (*i* = 1, 2, …, *N*_bind_) were distributed on the cargo’s surface in the following procedure: first, we discretized the cargo’s surface into *N*_all_ vertices (Fig. S2). The vertex number *N*_all_ was determined for each cargo so that the density of the vertex on cargo’s surface is conserved (Table S2). Then, we chose *N*_bind_ vertices out of *N*_all_ for the binding spots in a way that they conform to a particular surface distribution. We used two distinct surface distributions for the binding spots, namely uniform and nonuniform. The uniform distribution was generated to cover the whole side of the cargo below a specific latitudinal line (see Fig. S2 or schematics in Fig. 4 *B*). The nonuniform distribution was generated following the Kent distribution (79), whose probability density is expressed by the function below:

(19) $$f\left(x\right)=\frac{1}{c\left(\kappa ,\beta \right)}\text{exp}\left\{\kappa \gamma_{\left(1\right)}^{\text{T}}x+\beta \left[{\left(\gamma_{\left(2\right)}^{\text{T}}x\right)}^{2}-{\left(\gamma_{\left(3\right)}^{\text{T}}x\right)}^{2}\right]\right\},$$

where ***x*** is a three-dimensional unit vector showing the direction from the center of the cargo, *c*(*κ*, *β*) is a normalization constant, $\gamma_{\left(1\right)}^{\text{T}}$ is the mean direction, and $\gamma_{\left(2\right)}^{\text{T}},\gamma_{\left(3\right)}^{\text{T}}$ are the major and minor axes, which in this study are orthogonal to $\gamma_{\left(1\right)}^{\text{T}}$ and each other. Parameter *κ* signifies the concentration degree, and *β* determines the ellipticity of the distribution shape. We generated several nonuniform distributions by varying the parameter *κ* while keeping the parameter *β* to be 0.

In this study, we chose six different values for the number of binding spots *N*_bind_ (see Table S3). Hereafter, instead of explicitly showing the binding spot number *N*_bind_, we indicate their size using the parameter *S*, binding surface area, which signifies the surface area covered by the binding spots, i.e., *S* ~*N*_bind_/*N*_all_ × *π*$d_{\text{cargo}}^{2}$. The six cases in Table S3 correspond to the surface area ratio *S*/*S*^∗^ = 0.0, 0.2, 0.4, 0.6, 0.8, and 1.0, where *S*^∗^ is the reference value given as the entire surface area of a 20-nm-diameter cargo.

### Numerical calculations

Because the boundary condition and the external potential have irregular geometries, we solved the MDE using numerical methods. First, we decomposed the MDE in the direction of the contour variable *s* by applying the finite difference approximation of a backward Euler scheme. Then, we solved the remaining spatial problem by the finite element method (FEM). We discretized the space for the FEM using unstructured tetrahedral mesh, and we applied the P1 Lagrange element over the mesh to define the finite-dimensional functional space. We used the FEM solver provided by FEniCS project (80) to solve our FEM problem.

Our model description, which approximates the FG-Nups as continuous Gaussian chains, assumes its approximation validity above the spatial length of the reference Kuhn length *b* = 0.86 nm. To maintain the length scale of the approximation, we set the maximal volume of the tetrahedron mesh as *v*_tet_ = 0.1 nm^3^, which yields the edge size of the regular tetrahedron being 0.94 nm ~*b*. We also set the contour variable step size as Δ*s* = 0.4 so that the product of it with *b* becomes less than *b*, i.e., *b*Δ*s* < *b*.

### Critical diameter of the cargo

We calculated the free energy with and without a cargo inside the NPC, denoted by *F*_cargo_ and *F*_empty_, respectively, and analyzed the free energy difference between them, Δ*F* = *F*_cargo_ − *F*_empty_. The difference between *F*_cargo_ and *F*_empty_ stems from the change in the boundary condition and the addition of the interaction potential. When the cargo features binding spots, the free energy *F*_cargo_ varies depending on the orientation of the cargo. In that case, we calculated the averaged free energy ${\bar{F}}_{\text{cargo}}$ and the corresponding free energy difference $\text{Δ}\bar{F}={\bar{F}}_{\text{cargo}}$ − *F*_empty_ over the rotational freedom, that is, *θ* = *pπ*/5 (*p* = 0, 1, …, 19) in latitudinal direction and *φ* = *mπ*/10 (*m* = 0, 1, …, 10) in longitudinal direction.

**Figure 2:**
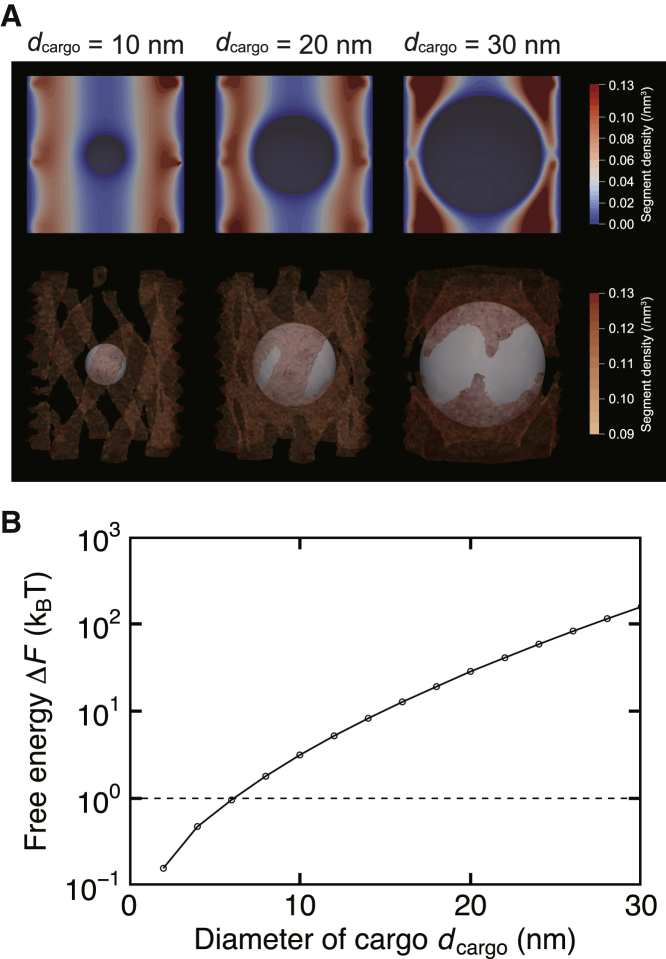
Free energy change induced by inserting an inert cargo into the NPC. (*A*) The mean segment density of FG-Nups with different cargo diameters *d*_cargo_. The cross-sectional distribution at the center of the pore (*top*) and the three-dimensional distribution (*bottom*) are shown. Visualized in the three-dimensional distribution are density values above a certain threshold. (*B*) The relation between the cargo diameter *d*_cargo_ and the free energy difference caused by the cargo insertion, Δ*F* = *F*_cargo_ − *F*_empty_. The dashed line indicates Δ*F* = *k*_B_*T*, below which cargoes can pass through the NPC without an external input.

**Figure 3:**
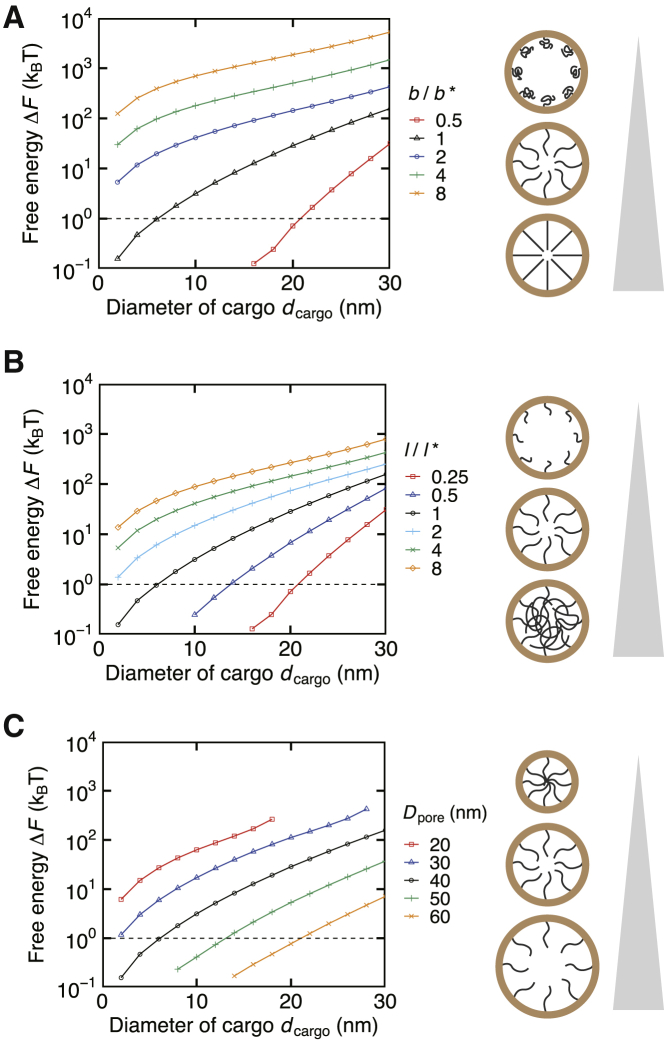
Effect of the NPC’s physical properties on the size-dependent selectivity. The relation between the cargo diameter *d*_cargo_ and the free energy difference induced by inserting an inert cargo, Δ*F* = *F*_cargo_ − *F*_empty_, is shown with different (*A*) Kuhn length *b* of FG-Nups, (*B*) total length *l* of FG-Nups, and (*C*) nuclear pore diameter *D*_pore_. Asterisked parameters, $b^{*}$ = 0.86 nm and $l^{*}$ = 180 nm, indicate the reference values. Considering the precision limit of our calculation, the data below 10^−1^ are removed from the plot. The dashed line indicates Δ*F* = *k*_B_*T*, below which cargoes can pass through the NPC without an external input. The schematics show the effect of each parameter on the overall NPC geometry.

We assumed that the free energy difference being less than the thermal energy, Δ*F* (or $\text{Δ}\bar{F}$) ≤ *k*_B_*T*, indicates that the cargo can get into the NPC by the thermal fluctuation. Supposing that the free energy difference Δ*F* (or $\text{Δ}\bar{F}$) depends on the cargo diameter *d*_cargo_ and that its value goes over *k*_B_*T* only once while increasing *d*_cargo_, we defined the cargo’s critical diameter $d_{\text{cargo}}^{\text{∗}}$ as the one yielding Δ*F* (or $\text{Δ}\bar{F}$) = *k*_B_*T*. The critical diameter $d_{\text{cargo}}^{\text{∗}}$ was calculated as follows; we first calculated the free energy difference Δ*F* (or $\text{Δ}\bar{F}$) with different cargo diameters *d*_cargo_. The sampling range of the cargo diameter *d*_cargo_ was set from 2 to 38 nm with the interval of Δ*d*_cargo_ = 2 nm. Then, we plotted the relation between the free energy difference Δ*F* (or $\text{Δ}\bar{F}$) and the cargo diameter *d*_cargo_ and interpolated them linearly. We identified the intersection between the interpolated data and the horizontal line Δ*F* (or $\text{Δ}\bar{F}$) $=k_{\text{B}}T$, and marked the intersection as the critical diameter $d_{\text{cargo}}^{\text{∗}}$. It should be noted that although Figs. 2 and 3 show the data in a logarithmic scale, we employed the linear interpolation to calculate the critical diameter.

### Clustering degree of the binding spots

To characterize the geometrical feature of the binding spot distribution, we measured the clustering degree *p*_cluster_. The clustering degree *p*_cluster_ indicates the concentration of the binding spots around a specific point on the cargo’s surface, determined by the following procedure; we first calculated the distance between every binding spot and the tethered positions of FG-Nups. We then specified a pair having the minimal distance among all pairs, and the binding spot in the pair was defined as the “nearest-to-wall” point. We considered a region within the radius of *r*_cluster_ from the nearest-to-wall point, and counted the number of vertices *N*_nw, all_ and binding spots *N*_nw, bind_ in it. The cutoff distance *r*_cluster_ was set to be 10*b* = 8.6 nm, where *b* is the reference Kuhn length. We then calculated the clustering degree by dividing the number of binding spots by the number of vertices, i.e., *p*_cluster_ = *N*_nw, bind_/*N*_nw, all__._

## Results and discussion

This study was aimed at quantitatively evaluating the hypothesis that the conformational entropy created by FG-Nups can be the basis of the NPC’s selectivity. To this end, we made a mathematical model that evaluates the free energy of the system composed of flexible FG-Nups inside the modeled NPC boundary. Using our model, we calculated the free energy in presence and absence of the cargo inside the NPC and studied whether the spontaneous translocation of the cargo into the NPC is likely to happen. We investigated the effect of the cargo size and the interactions between FG-Nups and NTRs.

### Free energy was calculated using the statistical description of FG-Nups

We numerically calculated the free energy created by FG-Nups using Edward’s field theory of polymers (68,69). Instead of explicitly modeling individual conformation of FG-Nups as is done by other studies (52), we used the statistical description of FG-Nups using the field variables $\tilde{q}$(***r***, *s*) and *q*_c_(***r***, *s*). The use of the field variables reduced the computational cost and enabled us to conduct a large number of calculations with different physical parameters. Specifically, a large amount of output from our calculation made it possible to analyze the effect of the cargo size, the cargo’s orientation in the NPC, the distribution of the binding spots on the cargo’s surface, and so on.

Our model description is precise above the spatial scale of FG-Nup’s reference Kuhn length, *b* = 0.86 nm (24). Because FG-Nups are flexible and natively unfolded with their persistence length much smaller than their average total length, i.e., *b* ≪ *l* = 180 nm, our continuous approximation is valid as long as we focus on the length scale larger than *b*. The spatial length scale of the precision was maintained during the numerical calculation by carefully choosing the discretization variables (see Materials and methods). The result of the control calculation showed that our numerical model represents well the analytical solution in a spatial scale larger than the reference Kuhn length *b*. Also, our control calculation measured the maximal error ratio (Δ*q*/*q*_numerical_)_max_ (Table S1) with Δ*q* being the difference between the numerical and analytical solution. Based on the maximal error ratio (Δ*q*/*q*_numerical_)_max_, we estimated the reliable range of the free energy difference to be |Δ*F*| ≥ 0.1 *k*_B_*T* (see Supporting materials and methods).

In this study, we calculated the free energy difference with and without the cargo inside the NPC, Δ*F* = *F*_cargo_ − *F*_empty_, where *F*_cargo_ is the free energy with a cargo inside the NPC and *F*_empty_ is the one without a cargo inside. The difference between *F*_cargo_ and *F*_empty_ stems from the difference of the boundary condition and the interaction potential around the binding spots (see Materials and methods). The interaction potential describes the energetic gain through the bindings between FG-Nups and NTRs. The interactions between FG-Nups, i.e., the steric repulsion and the cohesiveness among different segments of FG-Nups, were not included in our model so that we can investigate the effect of FG-Nup’s conformational entropy independently. This assumption is consistent with the observation by Davis et al. (81), which showed that FG-Nups have a physical property of the ideal polymers, in which repulsive and attractive interactions offset each other.

### Entropic effect created a barrier against inert cargoes larger than 6 nm in diameter

First, we calculated the free energy difference caused by the insertion of inert cargoes. Inert cargoes lack hydrophobic interactions with FG-Nups, hence the potential *V*(***r***) was set to be 0 throughout the calculation domain. The mean segment density was higher around the NPC wall (Fig. 2 *A*), reproducing the characteristic distribution of FG-Nups without cohesiveness (32,38,52, 53, 54, 55, 56). The mean segment density changed by varying the cargo size; although small cargoes did not perturb the density distribution appreciably, larger cargoes limited the space available to FG-Nups and altered the density distribution significantly. The notable change in the density distribution was accompanied by the change in system’s free energy (Fig. 2 *B*). The free energy increased by inserting the inert cargo and the resulting free energy difference Δ*F* increased monotonically with the cargo size, which is consistent with the free energy landscape calculated by Tagliazucchi et al. (53). By replotting the free energy difference Δ*F* as a function of the free volume available to FG-Nups, *v* = $\pi D_{\text{pore}}^{2}h_{\text{pore}}/4-\pi d_{\text{cargo}}^{3}$/6, we observed the convexity of the free energy (Fig. S3), which ensures the thermodynamical validity of our model.

The change in free energy caused by small cargoes was less than the thermal energy (Δ*F* < *k*_B_*T*), whereas the one of large cargoes was high enough (Δ*F* ≫ *k*_B_*T*) to block their entry into the pore. The critical diameter of the cargo, which yields Δ*F* = *k*_B_*T*, was calculated as $d_{\text{cargo}}^{\text{∗}}$ ~6 nm. The critical diameter matched well with the experimentally observed upper limit for the passive diffusion (5–9 nm (6, 7, 8)). It was also close to the critical diameter calculated by the coarse-grained molecular dynamics model by Ghavami et al. (5 nm (33)). It is interesting that our model did not include any cohesiveness between FG-Nups but still reproduced the critical diameter that was remarkably close to the experimentally and computationally observed values. Although there are various models proposed for the mechanism of NPC’s selectivity, we showed here that the entropic effect alone can create a practical barrier that selects molecules in a size-dependent manner.

To further compare our calculation results with experimental data, we estimated the mean force to insert the cargo into the NPC (Fig. S1). The estimated force (<200 pN) was in the same order as the one measured experimentally (<500 pN (42)), implying that the physical scale of our model is appropriate. The observation that the force estimated by our model is smaller than the one measured experimentally implies that the cohesiveness and the excluded volume effect of FG-Nups play a role in increasing the stiffness of NPC’s central plug.

We also investigated the effect of cargo’s position inside the NPC (Fig. S4). We observed only minor changes in free energy while vertically shifting the cargo within the pore. The lateral shift changed the free energy by less than 30 *k*_B_*T*, and the free energy was minimal at the center of the pore. This result implies that the inert cargo is likely to pass through the NPC along the central axis to minimize the free energy (37,53).

### Size-dependent selectivity changed depending on the Kuhn length of FG-Nups, total contour length of FG-Nups, and the nuclear pore diameter

Next, we investigated the effect of the physical properties of the NPC, namely the Kuhn length of FG-Nups *b*, the total contour length of FG-Nups *l*, and the pore diameter *D*_pore_ (Figs. 3 and S5). The free energy increased by increasing the Kuhn length *b*, increasing the contour length *l*, and decreasing the pore diameter *D*_pore_, respectively. These results were consistent with the geometrical insights arising from the change in each parameter (see schematics in Fig. 3). It is noteworthy that the size-dependent selectivity reproduced by our reference case was not conserved while changing these parameters. Instead, the critical diameter shifted drastically, and, in some cases, the free energy function did not even cross the line Δ*F* = *k*_B_*T*. This result indicates that for the entropy barrier hypothesis (or the virtual-gate model) to be quantitatively functional, fine tuning of the physical parameters is required, and that the natural NPC intrinsically contains the parameter set that is best to reproduce the size-dependent selectivity.

It is considered that the features regarding FG-Nups (Kuhn length *b* and contour length *l*) are evolutionarily conserved among different species (82, 83, 84, 85, 86). Given the sensitivity of the free energy to these physical parameters, their conservation would be essential to the proper functioning of the NPC. On the other hand, it is believed that the nuclear pore diameter *D*_pore_ is alterable in action; the NPC increases its pore diameter when a mechanical force is applied to the nuclear membrane (87,88). Recent studies hypothesized that the NPC’s dilation promotes molecular translocations from the cytoplasm to the nucleus (89,90). According to our result, when the nuclear pore diameter *D*_pore_ changes from 40 to 60 nm, which is in the observed range of the pore dilation (9,89), the critical diameter $d_{\text{cargo}}^{\text{∗}}$ changed from 6 to 20 nm, i.e., an ~3.3-fold increase, which is large enough to support the hypothesis. The effect of the pore dilation was further investigated for attractive cargoes in the latter sections.

### Bindings between NTRs and FG-Nups lowered the free energy to overcome the entropic barrier

**Figure 4:**
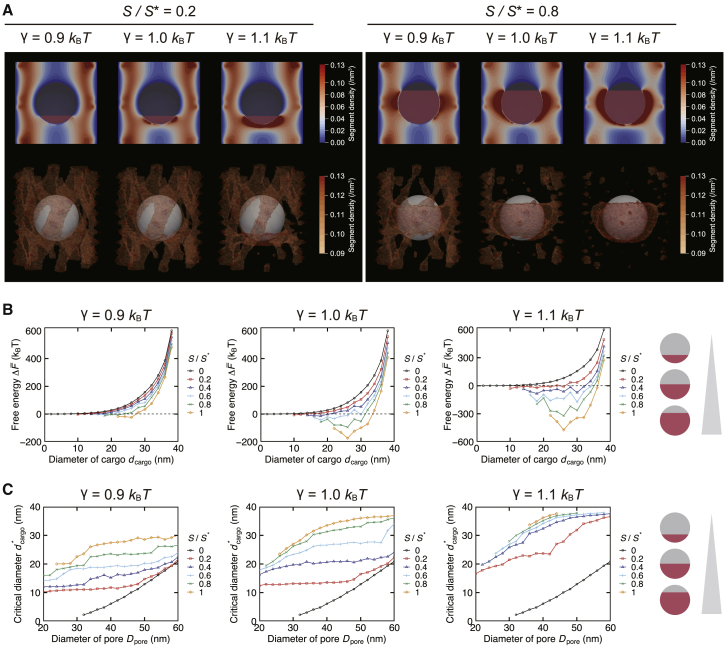
Free energy difference induced by inserting an attractive cargo into the NPC. Binding spots were distributed on the cargo’s surface following the uniform distribution. (*A*) The mean segment density of FG-Nups with different binding surface area *S* and interfacial energy *γ*. The cross-sectional distribution at the center of the pore (*top*) and the three-dimensional distribution (*bottom*) are shown. Visualized in the three-dimensional distribution are density values above a certain threshold. The cargo diameter *d*_cargo_ is 20 nm. The reference binding area $S^{*}$ is the surface area of a cargo whose diameter is 20 nm. (*B*) The relation between the cargo diameter *d*_cargo_ and the free energy difference accompanying the cargo insertion, $\text{Δ}\bar{F}={\bar{F}}_{\text{cargo}}$ − *F*_empty_. The effect of the interfacial energy *γ* and the binding surface area *S* are shown. The dashed line indicates $\text{Δ}\bar{F}$ = *k*_B_*T*. The schematics show how the binding spot distribution changes as we increase the binding area *S*. (*C*) The relation between the nuclear pore diameter *D*_pore_ and the critical diameter of the cargo $d_{\text{cargo}}^{\text{∗}}$, which signifies the maximal cargo size whose free energy difference is less than the thermal energy ($\text{Δ}\bar{F}$ < *k*_B_*T*).

Next, we calculated the free energy difference caused by the insertion of attractive cargoes. To model the interactions between NTRs and FG-Nups, we added the interaction potential *V*(***r***) in relation to the binding spots on the cargo. We first studied the effect of the uniformly distributed binding spots, in which we put all binding spots on one side of the cargo while leaving the other side totally noninteractive with FG-Nups (see Fig. S2 and schematics in Fig. 4 *B*). The uniform distribution can be seen as the classical model of the cargo-NTR complex, in which all binding spots are assumed to reside partially on the NTR surface.

The mean segment density map showed that the FG-Nups gathered around the binding spots (Fig. 4 *A*). As a result of the segment gathering, the relative density of the mean segment around their tethering spots decreased, implying the potential loss of their conformational entropy. This trend was qualitatively similar to the density map obtained by the homopolymer models of FG-Nups (53,55). The spatial inhomogeneity provoked by the interaction potential was further enhanced when we increased the interfacial energy *γ* and the binding surface area *S*.

Addition of the interaction potential resulted in the overall free energy reduction (Fig. 4 *B*). Compared with the case of inert cargoes, the free energy cost imposed to attractive cargoes became smaller, and for some cases, it even fell into negative, similarly to the results by Tagliazucchi et al. (53). The change in free energy was controlled by the interfacial energy *γ* and the binding surface area *S*; larger *γ* and larger *S* values resulted in smaller free energy, respectively. When the interfacial energy *γ* or the surface area *S* was large enough, the free energy changed nonmonotonically with the cargo size *d*_cargo_. This is likely because increasing the cargo size makes it easier for FG-Nups to reach the binding spots but reduces the available space for FG-Nups. The free energy decreased roughly linearly to the binding surface area *S* (Fig. S6). It is often the case that the linear relation between the binding spot number and the energetic gain is assumed (59), so our result showed that this assumption is appropriate when the binding spots are distributed uniformly. However, as we discussed in the later section, this was not the case for nonuniform distributions.

In most cases, we observed a significant increase in the cargo’s critical diameter $d_{\text{cargo}}^{\text{∗}}$ compared with the inert cargo cases (Fig. 4 *C*). When the interfacial energy *γ* and the surface area *S* were large enough, the critical diameter $d_{\text{cargo}}^{\text{∗}}$ approached the pore diameter *D*_pore_. This is consistent with the experimental observation that the maximal size of the transportable attractive cargo is as large as the nuclear pore size (9). To investigate the effect of the pore dilation, we calculated the critical diameter $d_{\text{cargo}}^{\text{∗}}$ with different size of the nuclear pore *D*_pore_. As expected, the pore dilation resulted in the increment of the critical diameter $d_{\text{cargo}}^{\text{∗}}$ (Figs. 4 *C*, S5, and S7). It should be noted that because the critical diameter of attractive cargoes was relatively large even when the pore size was small, the influence of the pore dilation was rather moderate compared with the inert cargo cases. This observation implies that the pore-size-dependent molecular transportation (89) is more effective on inert cargoes, whereas the transport rate of attractive cargoes is maintained regardless of the pore size.

### Clustered binding spots lowered the free energy

**Figure 5:**
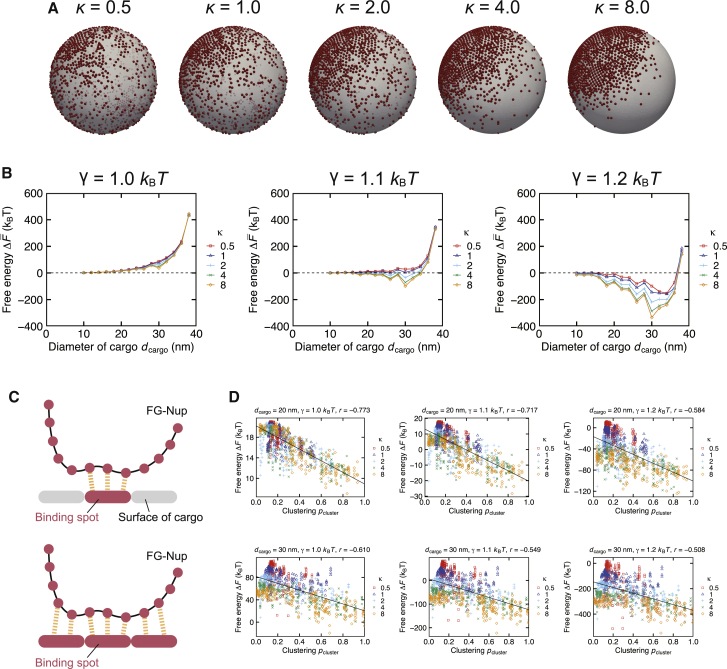
Effect of the binding spot distribution on the free energy. (*A*) The binding spot distribution following Kent distribution with different concentration parameter *κ*. The red point indicates the binding spot. Larger concentration parameter *κ* signifies more clustered distribution. (*B*) The relation between the cargo diameter *d*_cargo_ and the free energy difference accompanying the cargo insertion, $\text{Δ}\bar{F}={\bar{F}}_{\text{cargo}}$ − *F*_empty_. The effect of *κ* became more evident with larger interfacial energy *γ*. The dashed line indicates $\text{Δ}\bar{F}$ = *k*_B_*T*. (*C*) Schematics explaining the effect of binding spot clustering. When many binding spots are located around the same spot, subsequent segments of FG-Nups are locally bound to the cargo’s surface, which yields the energetic stability of the system. (*D*) The relation between the clustering degree *p*_cluster_ and the free energy difference Δ*F*. Each point indicates the data for a specific orientation of the cargo. Plotted by the black line is the linear regression of the data. The parameter *r* indicates the Pearson correlation coefficient.

Finally, we investigated the effect of the binding spot distribution on the free energy. In contrast to the previous section, where the binding spots were placed uniformly on one side of the cargo, we considered circumstances in which binding spots were nonuniformly distributed over the cargo’s surface. This model represents the idea that FG-Nups can interact with any hydrophobic surface on the cargo, including noncanonical spots outside of the NTR, which is distributed all over the cargo’s surface (48). We arranged the binding spots to follow the Kent distribution (79) (see Materials and methods) with different concentration parameter *κ*. Smaller *κ* values generated widely scattered binding spots, whereas larger *κ* values lead to a concentrated patch of the interaction site (Fig. 5 *A*). When the parameter *κ* was large enough, the distribution was close to the uniform distribution studied in the previous section. To focus on the effect of the distribution, we kept the total binding surface area constant, i.e., *S*/$S^{*}$ = 0.2, where $S^{*}$ is the total surface area of the 20-nm-diameter cargo.

Our results showed that the higher *κ* values, i.e., more concentrated binding spot distribution, yielded the lower free energy (Fig. 5 *B*). The difference in free energy became more evident as we increased the interfacial energy *γ*. When *γ* = 1.1 *k*_B_*T*, the free energy with *κ* = 0.5 was positive for all sizes of the cargo, but it showed negative values when *κ* ≥ 1.0 in a specific range of the cargo size, inducing the significant increase in the cargo’s critical diameter $d_{\text{cargo}}^{\text{∗}}$ (Table S4). These free energy differences were generated solely by the binding spot distribution instead of the binding surface area, implying that the geometrical factors play the important role on determining the cargo’s transportability.

We hypothesized that the decrease in free energy is due to the clustering of the binding spots. When the parameter *κ* is large enough, binding spots are clustered around the specific spot on the cargo. Once a segment of FG-Nup is attracted to such a spot, the subsequent segments are also bound there locally because the binding spots are neighboring to each other (Fig. 5 *C*). This consecutive binding of the subsequent segments energetically stabilizes the system, which effectively decreases the free energy. To support our hypothesis, we calculated the clustering degree *p*_cluster_, which signifies how many binding spots are located in the neighborhood of a specific spot (see Materials and methods). We calculated the clustering degree *p*_cluster_ for each orientation of the cargo and compared it with the free energy (Figs. 5 *D* and S8–S10). There is a negative correlation between the clustering degree *p*_cluster_ and the free energy, except when the cargo diameter is *d*_cargo_ = 38 nm. The exceptional case can be seen as a calculation error considering that there is only a 1 nm space between the pore wall and the cargo, which is close to the precision limit of our calculation. The negative correlation between the clustering degree and the free energy indicates that densely arranged binding spots contribute to decreasing the free energy.

Our hypothesis that clustered binding spots lowers the free energy is consistent with the observation from the molecular dynamics simulations by Isgro et al. (78,91). They showed that attractive cargoes such as importin-*β*, NTF2, and Cse1p contain multiple binding spots in close proximity to each other, whereas inert cargoes such as Kap60p have binding spots widely spread across the protein’s surface. They suggested that the density of the binding spots or the clustering degree, as well as the total number of the binding spots, determines whether the cargo is inert or attractive. The coarse-grained model of Ghavami et al. (33) also indicated that the potential of mean force became lower when they decreased the spacings between the binding spots. More recently, Davis et al. (92) studied the dynamics of the binding interaction between the attractive cargo and FG-Nups using a coarse-grained molecular dynamics simulation. They showed that when binding spots were distributed on one half of the cargo’s surface, the dissociation constant between FG-Nups and the cargo became significantly lower compared with when binding spots were distributed all over the cargo’s surface. All of these observations from the preceding studies are consistent with our calculation result, suggesting that geometrical factors such as the binding spot distribution are important in determining the selective nature of the NPC.

## Conclusions

In this study, we developed a theoretical and computational model to calculate the free energy of the NPC. Our results showed that the entropic barrier of FG-Nups was high enough (≫*k*_B_*T*) to effectively block the entry of large cargoes, whereas it was lower than the thermal energy (<*k*_B_*T*) for small cargoes with diameters less than 6 nm. This significant difference in the free energy barrier suggests that the size-dependent selections of inert cargoes can occur purely by the entropic effects. Indeed, this trend appeared only for some specific parameter sets, indicating the importance of physical features of FG-Nups, such as their flexibility and contour length, being evolutionarily conserved. On the other hand, attractive cargoes effectively lowered the free energy by bindings with FG-Nups. The energetic gain by each binding event was as small as ~*k*_B_*T*, but it was large enough to offset the entropic barrier of FG-Nups. Our results implied that the argument of the virtual-gate model, or the entropic barrier hypothesis, is quantitatively reasonable to explain the NPC’s selectivity. It should be noted that our calculations were performed based on the assumption of no mutual connectivity or cohesiveness among FG-Nups, focusing on the effect of the conformational entropy. In the case in which the intermolecular connectivity of FG-Nups dominates the system, other models, such as the selective phase model, would appear more suitable to explain the NPC’s selectivity.

Based on the entropic barrier assumption, we estimated the critical diameter of the cargo, which signifies the maximal cargo size that can pass through the NPC. For inert cargoes, the critical diameter was 6 nm, whereas for attractive cargoes, it increased up to the nuclear pore size. The nuclear pore dilation worked effectively to increase the critical diameter of both inert and attractive cargoes. Although the effect of the nuclear pore dilation was significant for inert cargoes, for which the transition of the pore size from 40 to 60 nm resulted in the critical diameter shift from 6 to 20 nm, the effect was rather moderate for attractive cargoes given that their critical diameters were already large for small pores. This suggests that the nuclear pore dilation is an effective way to lower the entropic barrier, especially for inert cargoes, and that a cell can regulate the size limit of the transportable cargoes by adjusting the nuclear pore diameter.

Another implication from our study is that the surface distribution of the binding spots on attractive cargoes changes the system’s free energy. Specifically, we showed that the free energy became smaller when the binding spots were clustered around a particular point on the cargo. We highlighted the importance of the binding spot distribution, as well as the interfacial energy and their surface area, as a factor to characterize the passage ability of attractive cargoes. Our study suggested that modulating geometrical factors such as the binding spot distribution has the potential to design cargoes that can efficiently pass through the NPC.

## Author contributions

A.M. and M.R.K.M. conceived the project and designed the computational experiments. A.M. developed the model and performed the simulations. A.M. and M.R.K.M. analyzed the data and wrote the manuscript.

## Supporting material

Document S1. Supporting materials and methods, Figs. S1–S10, and Tables S1–S4

Document S2. Article plus supporting material
