# Supplementary material for: Free energy calculations shed light on the nuclear pore complex’s selective barrier nature
Source: Biophys J. 2021 Jul 31;120(17):3628–40. doi: 10.1016/j.bpj.2021.07.025 (PMC8456294; doi:10.1016/j.bpj.2021.07.025)
Supplement: Document S1. Supporting materials and methods, Figs. S1–S10, and Tables S1–S4 [file mmc1.pdf]

Biophysical Journal, Volume *120*

Supplemental information

Free energy calculations shed light on the nuclear pore complex's selective barrier nature

Atsushi Matsuda and Mohammad R.K. Mofrad

Supplemental Material

Free Energy Calculations Shed Light on the Nuclear Pore Complex's Selective Barrier Nature

Atsushi Matsuda[1] and Mohammad R. K. Mofrad[1,2,*]

[1] Molecular Cell Biomechanics Laboratory, Departments of Bioengineering and Mechanical Engineering, University of California, Berkeley, CA 94720
[2] Molecular Biophysics and Integrative Bioimaging Division, Lawrence Berkeley National Laboratory, Berkeley, CA 94720
[*]Correspondence: mofrad@berkeley.edu

Derivation of the modified diffusion equation

Here we show the Green function (Eq. 5) satisfies the modified diffusion equation (MDE, Eq. 14). You can find more discussions on the MDE in (1, 2). We consider $G(\boldsymbol{r}, \boldsymbol{r}', s + \Delta s)$ for infinitesimal Δs. Using the Markov nature of the Green function,

$$G(\boldsymbol{r}, \boldsymbol{r}', s + \Delta s) = \int_\Omega \mathrm{d}\boldsymbol{r}''\, G(\boldsymbol{r}, \boldsymbol{r}'', \Delta s) G(\boldsymbol{r}'', \boldsymbol{r}', s). \tag{S.1}$$

We assume that the potential term $V(\boldsymbol{R}(s))$ can be approximated to be constant for $s \in [0, \Delta s]$, then it follows,

$$G(\boldsymbol{r}, \boldsymbol{r}'', \Delta s) = \exp(-\beta \Delta s V(\boldsymbol{r})) \int_{\boldsymbol{R}(0)=\boldsymbol{r}}^{\boldsymbol{R}(\Delta s)=\boldsymbol{r}''} \mathcal{D}\boldsymbol{R} \exp(-\beta U_0[\boldsymbol{R}]). \tag{S.2}$$

Suppose that $\boldsymbol{R}(s)$ is not on the boundary for $s \in [0, \Delta s]$, then the functional integral of the Gaussian chain is written by (1),

$$\int_{\boldsymbol{R}(0)=\boldsymbol{r}}^{\boldsymbol{R}(\Delta s)=\boldsymbol{r}''} \mathcal{D}\boldsymbol{R} \exp(-\beta U_0[\boldsymbol{R}]) = \left(\frac{3}{2\pi b^2 \Delta s}\right)^{\frac{3}{2}} \exp\left(-\frac{3|\boldsymbol{r} - \boldsymbol{r}''|^2}{2b^2 \Delta s}\right) \equiv G_0(\boldsymbol{r} - \boldsymbol{r}'', \Delta s). \tag{S.3}$$

Therefore,

$$G(\boldsymbol{r}, \boldsymbol{r}', s + \Delta s) = \exp(-\beta \Delta s V(\boldsymbol{r})) \int_\Omega \mathrm{d}\boldsymbol{r}''\, G_0(\boldsymbol{r} - \boldsymbol{r}'', \Delta s) G(\boldsymbol{r}'', \boldsymbol{r}', s). \tag{S.4}$$

By expanding $G(\boldsymbol{r}'', \boldsymbol{r}', s)$ with respect to $\boldsymbol{\eta} \equiv \boldsymbol{r} - \boldsymbol{r}''$,

$$\begin{aligned} I &= \int_\Omega \mathrm{d}\boldsymbol{r}''\, G_0(\boldsymbol{r} - \boldsymbol{r}'', \Delta s) G(\boldsymbol{r}'', \boldsymbol{r}', s) \\ &= \int \mathrm{d}\boldsymbol{\eta}\, G_0(\boldsymbol{\eta}, \Delta s) G(\boldsymbol{r} - \boldsymbol{\eta}, \boldsymbol{r}', s) \\ &= \int \mathrm{d}\boldsymbol{\eta}\, G_0(\boldsymbol{\eta}, \Delta s) \left(1 - \sum_{\alpha=1}^{3} \eta_\alpha \frac{\partial}{\partial r_\alpha} + \frac{1}{2} \sum_{\alpha=1}^{3} \sum_{\beta=1}^{3} \eta_\alpha \eta_\beta \frac{\partial^2}{\partial r_\alpha \partial r_\beta}\right) G(\boldsymbol{r}, \boldsymbol{r}', s). \end{aligned} \tag{S.5}$$

Since

$$\int \mathrm{d}\boldsymbol{\eta}\, G_0(\boldsymbol{\eta}, \Delta s) = 1, \quad \int \mathrm{d}\boldsymbol{\eta}\, G_0(\boldsymbol{\eta}, \Delta s) \eta_\alpha = 0, \quad \int \mathrm{d}\boldsymbol{\eta}\, G_0(\boldsymbol{\eta}, \Delta s) \eta_\alpha \eta_\beta = \frac{b^2 \Delta s}{3} \delta_{\alpha\beta}, \tag{S.6}$$

the integral becomes

$$I = \left(1 + \frac{b^2 \Delta s}{6} \nabla_{\boldsymbol{r}}{}^2\right) G(\boldsymbol{r}, \boldsymbol{r}', s). \tag{S.7}$$

By expanding the both sides of Eq. S.4 in terms of Δs,

$$\left(1 + \Delta s \frac{\partial}{\partial s}\right) G(\boldsymbol{r}, \boldsymbol{r}', s) = (1 - \beta \Delta s V(\boldsymbol{r})) \left(1 + \frac{b^2 \Delta s}{6} \nabla_{\boldsymbol{r}}^2\right) G(\boldsymbol{r}, \boldsymbol{r}', s). \tag{S.8}$$

Comparing the first order of Δs in the above equation gives the MDE.

Derivation of the mean segment density

Here we derive the relation between the mean segment density $\rho(\boldsymbol{r})$ and the Green function $G(\boldsymbol{r}, \boldsymbol{r}', s)$ (Eq. 10). The mean segment density $\rho(\boldsymbol{r})$ is the ensemble average of the microscopic segment density $\hat{\rho}(\boldsymbol{r}, \{[\boldsymbol{R}_i]_{i=1...n}\})$, defined by

$$\hat{\rho}(\boldsymbol{r}, \{[\boldsymbol{R}_i]_{i=1...n}\}) = \sum_{i=1}^{n} \hat{\rho}_i(\boldsymbol{r}, [\boldsymbol{R}_i]) \equiv \sum_{i=1}^{n} \int_0^N \mathrm{d}s\, \delta(\boldsymbol{r} - \boldsymbol{R}_i(s)). \tag{S.9}$$

Our model assumes that FG-Nups do not interact with each other and that their conformational freedom independently contributes to the partition function. Thus, the ensemble average of $\hat{\rho}(\boldsymbol{r}, \{[\boldsymbol{R}_i]_{i=1...n}\})$ can be written as

$$\rho(\boldsymbol{r}) = \langle \hat{\rho}(\boldsymbol{r}, \{[\boldsymbol{R}_i]_{i=1...n}\}) \rangle = \sum_{i=1}^{n} \langle \hat{\rho}_i(\boldsymbol{r}, [\boldsymbol{R}_i]) \rangle\,. \tag{S.10}$$

The ensemble average of the single FG-Nup's segment density $\langle \hat{\rho}_i(\boldsymbol{r}, [\boldsymbol{R}_i]) \rangle$ is

$$\langle \hat{\rho}_i(\boldsymbol{r}, [\boldsymbol{R}_i]) \rangle = \frac{\int_\Omega \mathrm{d}\boldsymbol{r}' \int_{\boldsymbol{R}_i(0)=\boldsymbol{r}_{\perp,i}}^{\boldsymbol{R}_i(N)=\boldsymbol{r}'} \mathcal{D}\boldsymbol{R}_i \exp\left(-\beta U_0[\boldsymbol{R}_i] - \beta U_1[\boldsymbol{R}_i]\right) \int_0^N \mathrm{d}s\, \delta(\boldsymbol{r} - \boldsymbol{R}_i(s))}{\int_\Omega \mathrm{d}\boldsymbol{r}' \int_{\boldsymbol{R}_i(0)=\boldsymbol{r}_{\perp,i}}^{\boldsymbol{R}_i(N)=\boldsymbol{r}'} \mathcal{D}\boldsymbol{R}_i \exp\left(-\beta U_0[\boldsymbol{R}_i] - \beta U_1[\boldsymbol{R}_i]\right)} \tag{S.11}$$

The delta function $\delta(\boldsymbol{r} - \boldsymbol{R}_i(s))$ ensures that conformational curves that does not pass through $\boldsymbol{R}_i(s) = \boldsymbol{r}$ vanishes. By breaking the conformational curve $[\boldsymbol{R}_i]$ into two parts $[\boldsymbol{R}_i']$ and $[\boldsymbol{R}_i'']$ (1),

$$\begin{aligned}\langle \hat{\rho}_i(\boldsymbol{r}, [\boldsymbol{R}_i]) \rangle &= \frac{\int_0^N \mathrm{d}s \int_\Omega \mathrm{d}\boldsymbol{r}' \int_{\boldsymbol{R}_i'(0)=\boldsymbol{r}_{\perp,i}}^{\boldsymbol{R}_i'(s)=\boldsymbol{r}} \mathcal{D}\boldsymbol{R}_i' \int_{\boldsymbol{R}_i''(s)=\boldsymbol{r}}^{\boldsymbol{R}_i''(N)=\boldsymbol{r}'} \mathcal{D}\boldsymbol{R}_i'' \exp\left(-\beta U_0[\boldsymbol{R}_i'] - \beta U_1[\boldsymbol{R}_i']\right) \exp\left(-\beta U_0[\boldsymbol{R}_i''] - \beta U_1[\boldsymbol{R}_i'']\right)}{\int_\Omega \mathrm{d}\boldsymbol{r}' \int_{\boldsymbol{R}_i(0)=\boldsymbol{r}_{\perp,i}}^{\boldsymbol{R}_i(N)=\boldsymbol{r}'} \mathcal{D}\boldsymbol{R}_i \exp\left(-\beta U_0[\boldsymbol{R}_i] - \beta U_1[\boldsymbol{R}_i]\right)} \\ &= \frac{\int_0^N \mathrm{d}s \int_\Omega \mathrm{d}\boldsymbol{r}'\, G(\boldsymbol{r}_{\perp,i}, \boldsymbol{r}, s) G(\boldsymbol{r}, \boldsymbol{r}', N-s)}{\int_\Omega \mathrm{d}\boldsymbol{r}'\, G(\boldsymbol{r}_{\perp,i}, \boldsymbol{r}', N)}\end{aligned} \tag{S.12}$$

Estimation of the calculation error

We estimated the calculation error that stems from the finite element modeling. We constructed a problem that can be solved analytically, and by comparing the analytical and numerical solutions, we estimated the upper bound of the calculation error.

We first set the calculation domain, which is a cubic space having one side of length L. The side length L was set to be 40 nm, which is in the same order of our NPC boundary. We then discretized the domain using the tetrahedron mesh. The maximum volume of the tetrahedron mesh was set to be 0.1 nm^3, which is the same discretization scheme as our NPC model. We solved the modified diffusion equation (Eq. 14) in this domain with the external potential term being zero, $V(\boldsymbol{r}) = 0$. The six faces of the cube was set to be the Dirichlet boundaries (Eq. 16). We adopted the uniform initial condition, $q(\boldsymbol{r}, 0) = 1$, resembling the end-segment distribution $\tilde{q}(\boldsymbol{r}, 0)$. Parameters for the calculations were set to be the same as the NPC model (Kuhn length $b = 0.86$ nm, total length of FG-Nup $l = 180$ nm, and one step of the contour position variable $\Delta s = 0.4$).

We sampled the numerical solution at points $(Li/20, Lj/20, Lk/20)$ where i, j, and k are integers in the range of $[1, 19]$. We calculated the difference between the analytical and numerical solutions $\Delta q = \left|q_{\mathrm{analytical}} - q_{\mathrm{numerical}}\right|$, and evaluated its average and maximum values (Table S1). Free energy F is calculated by the logarithmic summation of the end-segment distribution $\tilde{q}(\boldsymbol{r}, N)$, and hence, the free energy difference $\Delta F = F_{\mathrm{cargo}} - F_{\mathrm{empty}}$ contains the calculation error, which is in the order of $\ln(1 + \Delta q/q)$. By using the maximum error ratio in Table S1, we estimated the precision of the free energy difference to be $|\Delta F| > 0.1$ ($k_{\mathrm{B}}T$).

Table S1: Error between the analytical and numerical solutions $\Delta q = \left|q_{\mathrm{analytical}} - q_{\mathrm{numerical}}\right|$.

Average error	$(\Delta q)_{\mathrm{average}}$	0.000921
Maximum error	$(\Delta q)_{\mathrm{max}}$	0.002322
Average error ratio	$(\Delta q/q_{\mathrm{numerical}})_{\mathrm{average}}$	0.003149
Maximum error ratio	$(\Delta q/q_{\mathrm{numerical}})_{\mathrm{max}}$	0.056566

Estimation of the force inserting cargo into the NPC

We estimated the force needed to insert an inert cargo into the NPC. We first estimated the pressure $p(d_{\text{cargo}})$ to insert the cargo of the diameter d_{cargo} by $p(d_{\text{cargo}}) = (F(d_{\text{cargo}}) - F(d_{\text{cargo}} - \Delta d_{\text{cargo}}))/(v_c(d_{\text{cargo}}) - v_c(d_{\text{cargo}} - \Delta d_{\text{cargo}}))$, where $F(d_{\text{cargo}})$ is the free energy, $v_c(d_{\text{cargo}}) = \pi d_{\text{cargo}}^3/6$ is the volume of a cargo, and $\Delta d_{\text{cargo}} = 2$ nm is the sampling interval. We then assumed the pressure $p(d_{\text{cargo}})$ is the average pressure to insert the cargo into the NPC, and estimated the insertion force by $f_{\text{insert}}(d_{\text{cargo}}) = p(d_{\text{cargo}}) \times \pi d_{\text{cargo}}^2$. The calculation result is shown in Fig. S1.

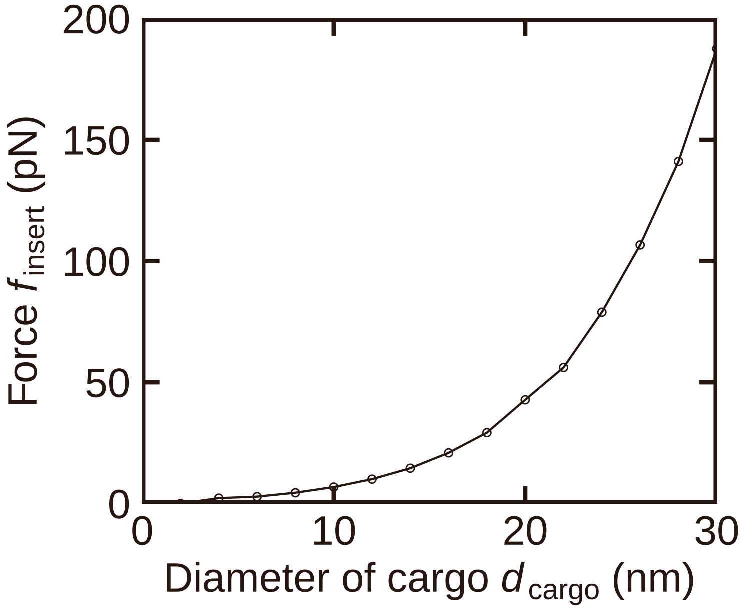

Figure S1: Average force to insert an inert cargo into the NPC. The force was estimated from the free energy difference between the different cargo size.

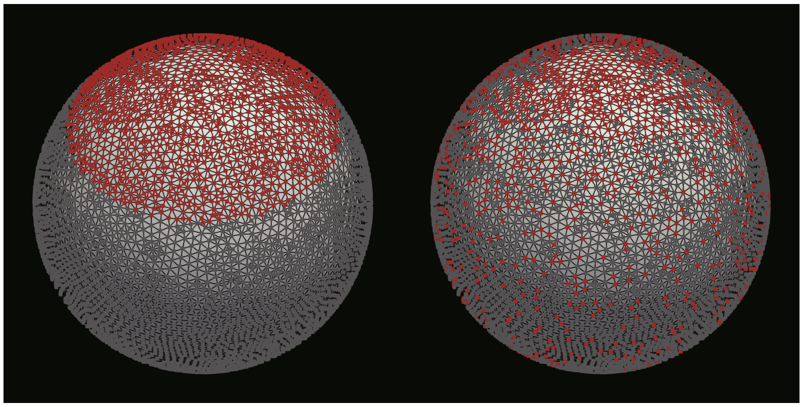

Figure S2: Uniform (left panel) and non-uniform (right panel) distributions of the binding spots on the cargo. We discretized the cargo's surface into N_{all} vertices, and chose N_{bind} binding spots among them. Binding spots and non-binding vertices are colored red and gray, respectively. The cargo's diameter and the binding surface area are $d_{\text{cargo}} = 20$ nm and $S/S^* = 0.2$ in both panels. The concentration parameter of the non-uniform distribution (right panel) is $\kappa = 1.0$.

Table S2: Number of vertices on cargoes. We determined the number of vertices N_{all} so that the surface area per vertex is conserved between different cargo diameter d_{cargo}.

Cargo diameter d_{cargo} (nm)	Number of vertices N_{all}	Surface area per vertex (nm^2)
2	77	0.163
4	289	0.174
6	642	0.176
8	1142	0.176
10	1773	0.177
12	2569	0.176
14	3468	0.178
16	4557	0.176
18	5782	0.176
22	8620	0.176
20	7130	0.176
24	10264	0.176
26	12042	0.176
28	13978	0.176
30	16051	0.176
32	18260	0.176
34	20618	0.176
36	23124	0.176
38	25735	0.176

Table S3: Relation between the binding surface area ratio and the number of binding spots. The reference surface area, S^*, indicates the entire surface area of a 20-nm-diameter cargo.

Binding surface area S/S^*	Number of binding spots N_{bind}
0.0	0
0.2	1426
0.4	2852
0.6	4278
0.8	5704
1.0	7130

Table S4: The critical diameter d^*_{cargo} of the attractive cargo having non-uniformly distributed. Data are shown in the unit of nanometer. The parameters γ and κ indicates the interfacial energy and the concentration parameter of the Kent distribution. When $\gamma = 1.0\ k_BT$ and $\kappa = 0.5, 1.0, 2.0, 4.0, 8.0$, or when $\gamma = 1.0\ k_BT$ and $\kappa = 0.5$, the free energy difference ΔF was positive for all diameters of the cargo, so d^*_{cargo} was not calculated.

	$\gamma = 1.0\ k_BT$	$\gamma = 1.1\ k_BT$	$\gamma = 1.2\ k_BT$
$\kappa = 0.5$	-	-	37
$\kappa = 1.0$	-	26	37
$\kappa = 2.0$	-	27	37
$\kappa = 4.0$	-	34	37
$\kappa = 8.0$	-	34	37

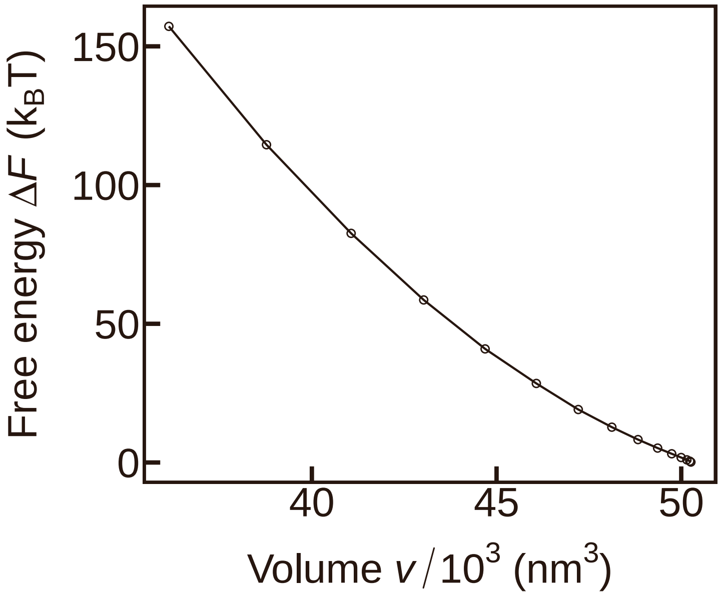

Figure S3: Free energy difference ΔF caused by the insertion of the inert cargo. The data shown in Fig. 2 were re-plotted as a function of the volume available to FG-Nups, $v = \pi D_{\mathrm{pore}}^2 h_{\mathrm{pore}}/4 - \pi d_{\mathrm{cargo}}^3/6$.

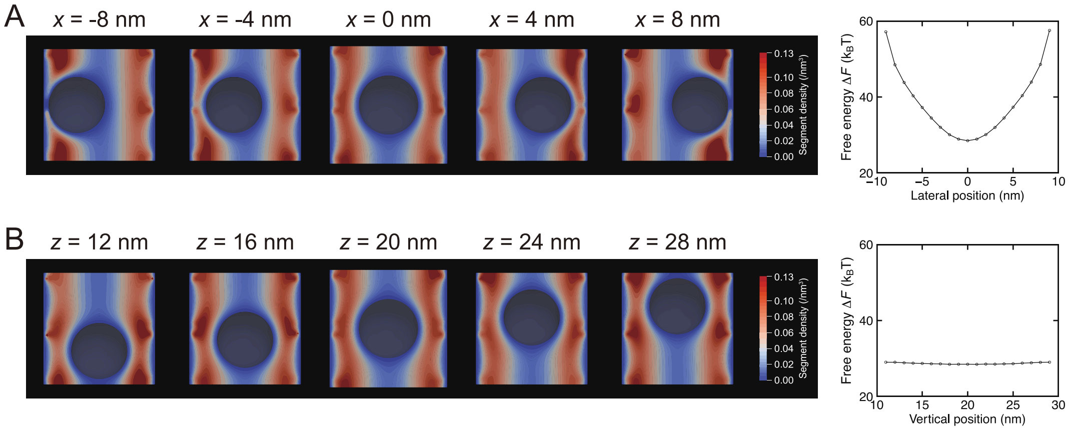

Figure S4: Effect of the cargo's position inside the nuclear pore. The free energy was calculated with different (A) lateral and (B) vertical position of the cargo. The left figures show the mean segment density cross-sectioned at the center of the pore. Red and blue color indicate higher and lower density. The right figures show the free energy difference ΔF, which signifies the difference with and without the cargo inside the pore ($\Delta F = F_{\mathrm{cargo}} - F_{\mathrm{empty}}$). The calculation was done with the inert cargo whose diameter was $d_{\mathrm{cargo}} = 20$ nm.

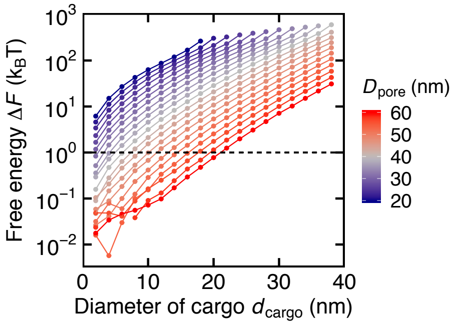

Figure S5: The free energy of the inert cargo with different diameter of the nuclear pore. The relation between the cargo's diameter d_{cargo} and the free energy difference ΔF with various diameter of the nuclear pore D_{pore} is shown. Indicated by the dashed line is $\Delta F = k_{\mathrm{B}}T$, below which the cargo can pass through the NPC. The critical diameter d^{*}_{cargo} in Fig. 4 (main text) denotes the intersection between the plotted line and the dashed line ($\Delta F = k_{\mathrm{B}}T$).

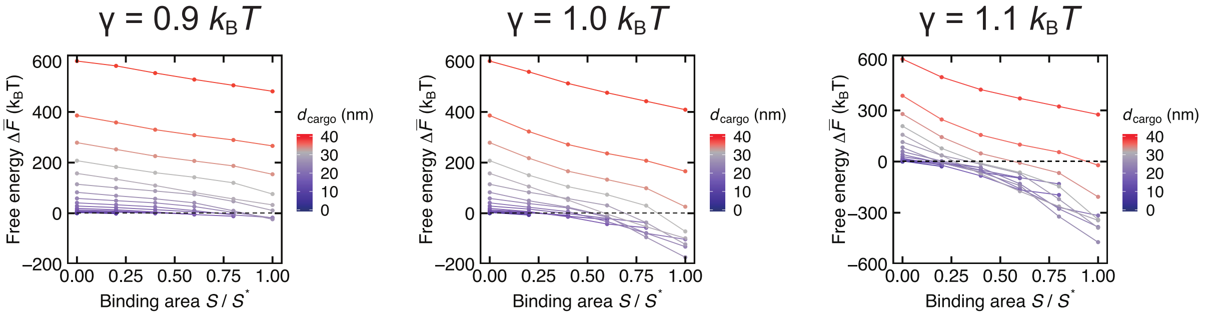

Figure S6: The relation between the free energy and the binding surface area. Data with the attractive cargo having uniformly distributed binding spots are shown (same data as Fig 4 B with binding surface area S/S^{*} on the horizontal axis). The size of the nuclear pore is $D_{\mathrm{pore}} = 40$ nm. The size of the cargo d_{cargo} is in the range between 2 and 38 nm, which is shown in the color legend. Dashed line indicates $\Delta\bar{F} = k_{\mathrm{B}}T$.

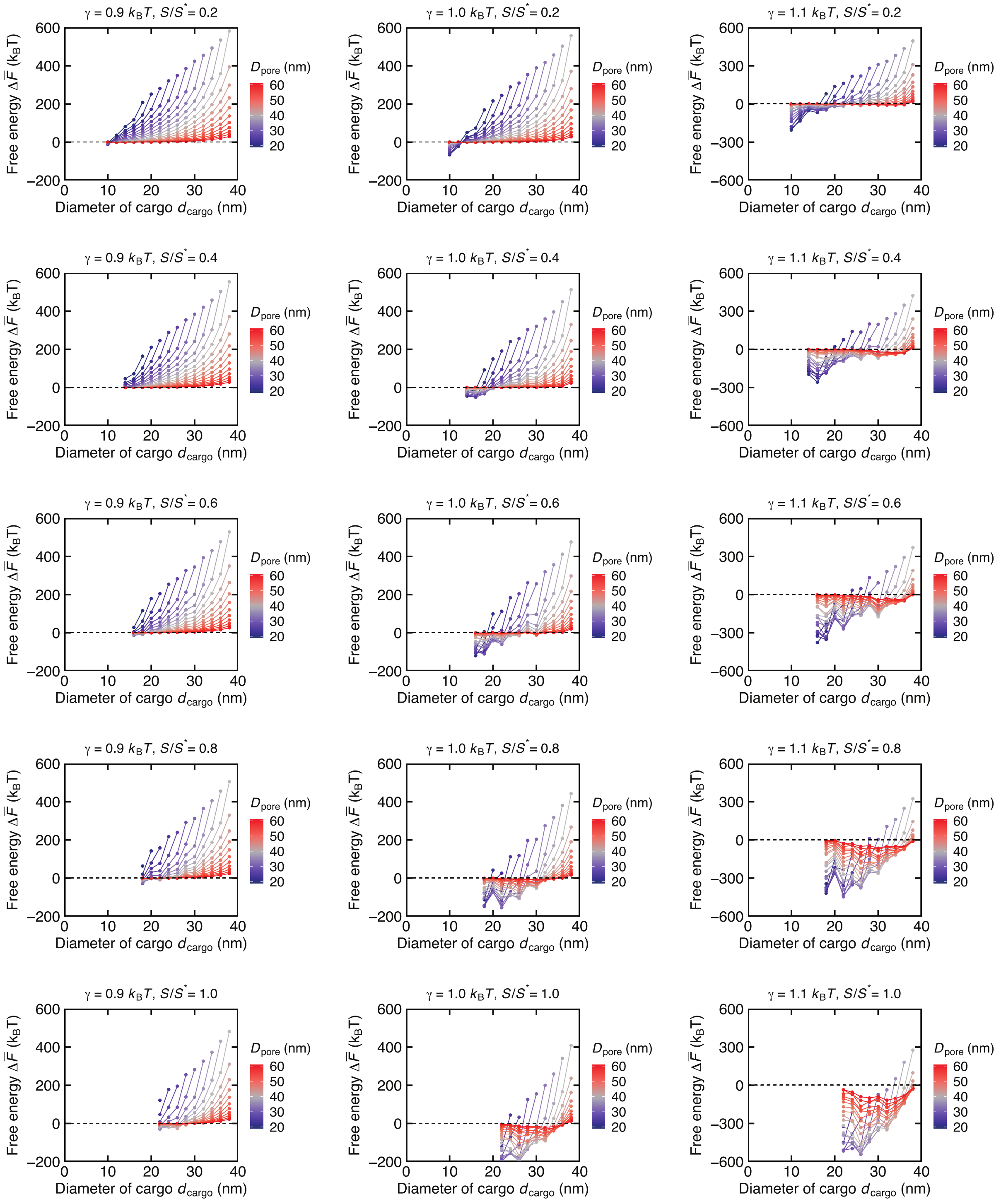

Figure S7: The free energy of the attractive cargo with different diameter of the nuclear pore. The relation between the cargo's diameter d_{cargo} and the free energy difference $\Delta\bar{F}$ with various diameter of the nuclear pore D_{pore} is shown. Each point signifies the average free energy with respect to all the orientations of the cargo. Binding spots were distributed uniformly below a longitudinal line of the cargo. The interfacial energy and the binding surface area are respectively denoted as γ and S. Indicated by the dashed line is $\Delta\bar{F} = k_B T$, below which the cargo can pass through the NPC. The critical diameter d^*_{cargo} in Fig. 4 (main text) denotes the intersection between the plotted line and the dashed line ($\Delta\bar{F} = k_B T$).

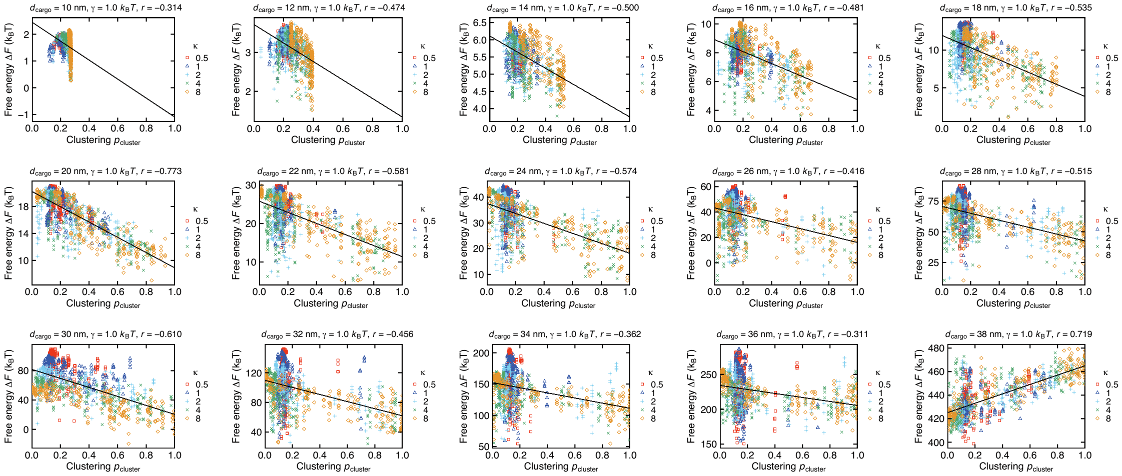

Figure S8: The relation between the clustering degree p_{cluster} and the free energy difference ΔF when the interfacial energy was $\gamma = 1.0 k_{\text{B}}T$. Data calculated for the attractive cargo having non-uniformly distributed binding spots are shown. Each point signifies the value of a specific orientation of the cargo. The cargo's diameter and the concentration parameter of the Kent distribution are indicated as d_{cargo} and κ. The clustering degree and the free energy showed the negative correlation except for the case of d_{cargo} = 19 nm. The correlation coefficient r is shown on top of the figure and the linear regression line is superposed on the data.

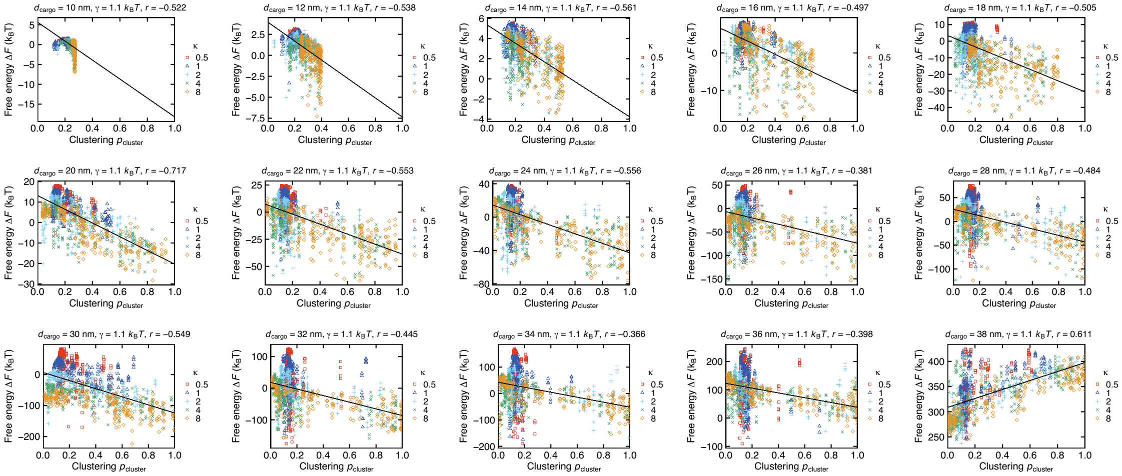

Figure S9: The relation between the clustering degree p_{cluster} and the free energy difference ΔF when the interfacial energy was $\gamma = 1.1 k_{\text{B}}T$. Data calculated for the attractive cargo having non-uniformly distributed binding spots are shown. Each point signifies the value of a specific orientation of the cargo. The cargo's diameter and the concentration parameter of the Kent distribution are indicated as d_{cargo} and κ. The clustering degree and the free energy showed the negative correlation except for the case of d_{cargo} = 19 nm. The correlation coefficient r is shown on top of the figure and the linear regression line is superposed on the data.

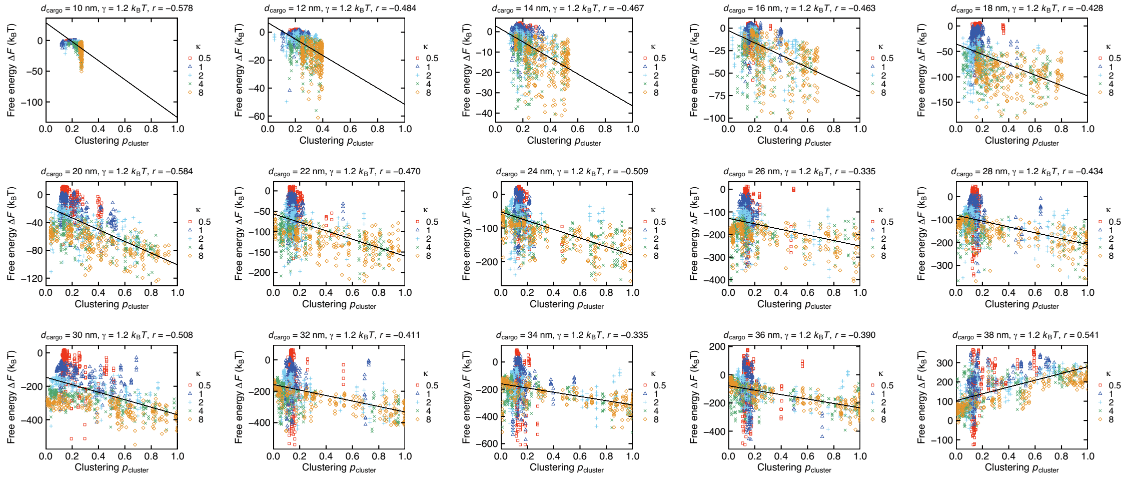

Figure S10: The relation between the clustering degree p_{cluster} and the free energy difference ΔF when the interfacial energy was $\gamma = 1.2k_BT$. Data calculated for the attractive cargo having non-uniformly distributed binding spots are shown. Each point signifies the value of a specific orientation of the cargo. The cargo's diameter and the concentration parameter of the Kent distribution are indicated as d_{cargo} and κ. The clustering degree and the free energy showed the negative correlation except for the case of $d_{\text{cargo}} = 19$ nm. The correlation coefficient r is shown on top of the figure and the linear regression line is superposed on the data.

REFERENCES

1. Freed, K. F., 1972. Functional Integrals and Polymer Statistics. *Advances in Chemical Physics* 22:1–128.

2. Doi, M., and S. F. Edwards, 1988. The theory of polymer dynamics. Oxford University Press.
